# Single nucleotide polymorphisms associated with benzimidazole resistance of the *β-tubulin* isotype 1 gene in *Ascaris lumbricoides* isolated in South Africa

**DOI:** 10.1016/j.bjid.2025.104556

**Published:** 2025-06-28

**Authors:** Teniel Ramkhelawan, Pragalathan Naidoo, Zilungile L. Mkhize-Kwitshana

**Affiliations:** a University of KwaZulu-Natal, Nelson R. Mandela School of Medicine, College of Health Sciences, School of Laboratory Medicine and Medical Sciences, Department of Medical Microbiology, KwaZulu-Natal, Durban, South Africa; b South African Medical Research Council (SAMRC), Division of Research Capacity Development, Cape Town, Western Cape, South Africa; c University of South Africa, College of Agriculture and Environmental Sciences, Biomedical Sciences Department of Life and Consumer Sciences, Johannesburg, South Africa

**Keywords:** *A. lumbricoides, β-tubulin* isotype 1 gene, Single nucleotide polymorphisms, Benzimidazole drug resistance, Treatment efficacy

## Abstract

**Background:**

Ascariasis is a parasitic infection caused by *Ascaris lumbricoides* and infects over 1.2 billion people worldwide. Benzimidazole (BZ) drugs remain the standard treatment in large-scale deworming programs globally. The prevalence of Single Nucleotide Polymorphisms (SNPs) in the *β-tubulin* gene of *A. lumbricoides* (F200Y, E198A and F167Y) is increasing due to the widespread use of BZ drugs.

**Aim:**

To investigate the prevalence of the above-mentioned SNPs in a South African adult population.

**Methods:**

This was a sub-study of the main cross-sectional study with participants (*n* = 414) who had been recruited from five public health clinics in the peri‑urban areas South of Durban, KwaZulu-Natal, South Africa. For the current study, a purposive selection of 20 stool samples that were positive for *A. lumbricoides* eggs was made. *A. lumbricoides* worm extracts (*n* = 4) were used as a positive control. Sanger sequencing and RFLP-PCR were used to identify the presence of mutations.

**Results:**

No mutations were detected, and all genotypes observed at codons F167Y, E198A and F200Y were the homozygous wild-type genotype.

**Conclusion:**

Although no mutations were found in this small study, the potential occurrence of mutations in a larger sample subset cannot be ruled out.

## Introduction

Soil-Transmitted Helminths (STH) are parasitic worms that are transmitted through contact with contaminated soil. Globally, the most common STH species that infect humans are *Trichuris trichiura,* Hookworms (*Ancylostoma duodenale* and *Necator americanus*) and *Ascaris* lumbricoides^1^^,^^2^ The global prevalence of STH infections is estimated to be around 24 %, with an estimated 1.5 billion people infected^3^^,^^4^

Ascariasis, the infection caused by *A. lumbricoides*, is the most common parasitic infection in humans, and around 1.2 billion people are estimated to be infected worldwide^5^ *A. lumbricoides* is the largest human intestinal parasite[5] and manifest in areas exposed to poverty and unsanitary living conditions^6^ Ascariasis is associated with malnutrition and several diseases,^7^ and impaired cognitive development and growth retardation in children^8^ Around 60,000 deaths per year are caused by *A. lumbricoides* infections^9^ In sub-Saharan Africa, an estimated 173 million people are infected with *A.* lumbricoides^10^

The World Health Organization (WHO) recommends the administration of deworming drugs to all susceptible populations (mainly school children)^11^ Large-scale deworming initiatives that seek to reduce the morbidity brought on by intestinal worms continue to use benzimidazole medications as the standard of treatment, namely Albendazole (a single dose of 400 mg taken orally) and Mebendazole (a single dose of 500 mg taken orally or two doses daily of 100 mg taken orally over three days)^9^

The target for these benzimidazole drugs in *A. lumbricoides* is the *β-tubulin gene*, where resistance is likely to develop due to high drug pressure, highlighting the need for surveillance systems to detect gene mutations associated with benzimidazole resistance^12^ However, the development of such tools is impeded by a lack of insight into the genes coding for the β-tubulin proteins, which are the principal targets of benzimidazole drugs^12^

To date, benzimidazole resistance has been linked to mutations in β-tubulin proteins, more specifically at codons 200, 167 or 19,8^13^ These mutations at codons 200, 167 and 198 are associated with benzimidazole resistance in a variety of animals[14–16] and conferred phenotypic resistance in the transgenic model organism *Caenorhabditis* elegans^16^ Nematodes usually encode multiple *β-tubulin* isotypes that are expressed at different stages during their life cycle^17^^,^^18^ One of the highly expressed isotypes, *β-tubulin* isotype 1, is commonly linked to resistance in parasitic nematodes^15^^,^^16^^,^^19^^,^^20^

Benzimidazole-resistant *A. lumbricoides* have SNPs at codons 200, 198 and 167 in the *β-tubulin gene* that alter the amino acid sequence of the tubulin protein, which causes the protein to be less sensitive to benzimidazole drugs. The most common SNPs that have been identified in the *β-tubulin* gene of *A. lumbricoides* that are associated with benzimidazole resistance are F167Y (TTC, TTT/Phenylalanine to TAC, TAT/Tyrosine)^21^ and F200Y (TTC/Phenylalanine to TAC/Tyrosine);[21] these SNPs cause amino acid substitution of phenylalanine to tyrosine. The least prevalent SNP, E198A (GAG, GAA/Glutamic acid to GCG, GCA/Alanine), causes an amino acid substitution of glutamic acid to alanine^21^^,^^22^

South Africa, as a signatory to the World Health Assembly (WHA) resolution 54.19 (May 2001), which calls for regular, synchronised treatment of worms in high-risk groups, implemented the first national deworming programme targeting school children in February 201,6^23^ The programme uses 500 mg single dose Mebendazole to deworm school-going children annually. There are two benzimidazoles registered in South Africa, Mebendazole, which is available over the counter and thus widely used, and Albendazole, which is a schedule 4 prescription drug^23^

It has been shown that SNPs in the *β-tubulin gene* of *A. lumbricoides* are associated with benzimidazole resistance, and the prevalence of these SNPs is increasing^21^^,^^22^^,^^24^, ^25^, ^26^ Although sub-Saharan Africa is highly endemic for ascariasis and large-scale deworming has been implemented in the region,^3^ there is a lack of studies focusing on the prevalence of these benzimidazole-resistant SNPs, particularly in South Africa. present study aimed to investigate the prevalence of the F200Y, E198A and F167Y SNPs in the *β-tubulin* isotype 1 gene of *A. lumbricoides* isolated from a South African population.

## Methods and materials

### Ethics approval

This study was approval by the Biomedical Research Ethics Committee (BREC) at the University of KwaZulu-Natal (BE351/19 and BREC/00,004,834/2022). The work described has been carried out in accordance with The Code of Ethics of the World Medical Association (Declaration of Helsinki) for experiments involving humans.

### Patient recruitment and study setting

This was a sub-study of the main cross-sectional study with participants (*n* = 414) who had been recruited from five public health clinics in the peri‑urban areas South of Durban, KwaZulu-Natal, South Africa. Details on the study setting and patient recruitment have been previously described by Mpaka-Mbatha et al. (2023)^27^ BMI measurements were made at the clinics and demographic qualitative data including history of deworming exposure, was collected through administration of structured interview questionnaires^27^

### Detection of A. lumbricoides infection

*A. lumbricoides* eggs and worms were detected in stool samples using the Kato-Katz quantification technique (Sterlitech: Auburn, Washington DC, USA) and a modified formol ether Mini Parasep® SF faecal parasite concentrator technique (Apacor Ltd., Wokingham, UK) followed by coproscopy. To increase the specificity and sensitivity of coproscopy, each participant donated two stool samples two to three days apart.

### Sample selection for β-tubulin isotype 1 gene SNP detection

From the main study, 137 of the 414 participants were infected with parasites, and 79 of the 137 had *Ascaris lumbricoides* eggs in stool. For the current study, a purposive selection of 20 stool samples that were positive for *A. lumbricoides* eggs was made. *A. lumbricoides* worm extracts (*n* = 4) were donated by Prof William Horsnell, Head of the Parasite Immune Regulation Laboratory, University of Cape Town for use as positive controls.

### DNA isolation

DNA was extracted from stool samples and worm extracts using the Quick-DNA™ Faecal/Soil Microbe Miniprep Kit (Zymo Research, United States, Catalogue number: D6010). The concentration and purity of the extracted DNA was assessed using the Nanodrop 2000 spectrophotometer (ThermoFisher Scientific, United States). The extracted DNA were stored at −20 °C for further genetic studies.

### Amplification of the β-tubulin isotype 1 gene and β-tubulin isotype 1 gene SNPs (F200Y, F167Y and E198A) of A. lumbricoides

The DreamTaq Green PCR Master Mix (Thermofisher Scientific, Catalogue number: K1081) and the Applied Biosystems 7500 PCR Detection System (Thermofisher Scientific) was used to amplify the 564 bp *β-tubulin* isotype 1 gene,^21^^,^^25^ and the 629 bp *β-tubulin* isotype 1 gene F200Y SNP,^21^ 608 bp *β-tubulin* isotype 1 gene F167Y SNP and 608 bp *β-tubulin* isotype gene E198A SNP[22,25] (*A. lumbricoides* worm extracts: *n* = 4 and study participants: *n* = 20).

Each PCR reaction contained 20 µL of DreamTaq Green PCR Master Mix (2X), 2 µL of each forward and reverse primers for the *β-tubulin* isotype 1 gene and its associated SNPs which was synthesized at Inqaba Biotechnical Industries (South Africa) (Table 1), 4 µL nuclease-free water and 2 µL of DNA extracted from worm extracts and stool samples. The PCR conditions were as follows: (i) Initial denaturation at 95 °C for 3 minutes, (ii) Followed by 35 cycles: denaturation at 95 °C for 30 seconds, annealing temperature specific for the *β-tubulin* isotype 1 gene and its associated SNPs (Table 1) for 30 seconds and elongation at 72 °C for 1 minute, and (iii) Final elongation at 72 °C for 10 minutes.

**Table 1:** Sequences of the published primers used to amplify the *β-tubulin* isotype 1 gene and the SNPs of *A. lumbricoides* in the study population (*n* = 20) published by Rashwan et al^25^ and Diwara et al.^26^.

Primer name	Forward primer sequence	Reverse primer sequence	Annealing Temperature ( °C)
*β -tubulin* isotype 1 gene	5′-CCAGCTGACGCACTCGCTTGG-3′	5′-ATGGTTGAGGTCTCCGTATGTG-3′	59 °C
F200Y	5′-GAACACCGATGAAACCTACTG-3′	5′-CAGTAGGTTTCATCGGTGTTC-3′	58.7 °C
F167Y	5′-GCTCGTACTCAGTTGTTCCATC-3′	5′-GATGGAACAACTGAGTACGAGC-3′	58.4 °C
E198A	5′-GAACACCGATGCAACCTTC −3′	5′-GAAGGTTGCATCGGTGTTC-3′	58.7 °C

Verification of the amplified PCR products was done using agarose gel electrophoresis. The amplified PCR products and DNA ladder were run on 2 % agarose gel containing 6 µL GelRed and visualised using the ChemiDoc™ XRS+ Molecular Imaging System (Bio-Rad). Amplified PCR products were stored at −20 °C until further genetic analyses.

### Sanger sequencing

The positive *β-tubulin* isotype 1 gene PCR amplicons (*A. lumbricoides* worm extracts: *n* = 4 and study participants: *n* = 20) were sequenced to verify the amplification of the gene of interest and to confirm the presence or absence of the SNPs at codons F167Y, F200Y and E198A using Sanger sequencing. Each amplicon was sequenced in both directions (forward and reverse) to cover the full-length *β-tubulin* isotype 1 gene. The sequencing was conducted using the BrilliantDye™ Terminator v3.1 Cycle Sequencing on an ABI3500XL genetic analyser (Thermo Fisher Scientific, MA, USA). The sequencing was performed at Inqaba Biotechnical Industries (South Africa). Ambiguous nucleotides in the ABI sequencing files were manually inspected and corrected (if necessary) on CHROMAS (Technelysium, Queensland, Australia). The forward and reverse sequences were aligned using the DNAMAN software (Lynnon Biosoft, California, United States). The identity of the edited sequences was confirmed using the National Centre for Biotechnology Information (NCBI) Basic Local Alignment Search Tool (BLAST).

### Detection of β-tubulin isotype 1 gene F200Y, F167Y and E198A SNPs of A. lumbricoides using RFLP- PCR

The genotyping of the F200Y, F167Y and E198A SNP PCR amplicons were performed using RFLP-PCR. The F200Y, F167Y and E198A PCR gene amplicons were each digested with the HpyCH4III, RsaI and BsmI restriction enzymes[22,25] (New England Biolabs), respectively (Table 2). Thereafter, the restriction digests were run on a 3 % agarose gel containing 6 µL of GelRed and visualised using the ChemiDoc™ XRS+ Molecular Imaging System (Bio-Rad). The expected fragment sizes for the homozygous wild-type genotype, heterozygous variant genotype and homozygous variant genotype for each of the *β-tubulin* isotype 1 gene F200Y,^25^ F167Y[22] and E198A[22] SNPs is summarized in Table 2.

**Table 2:** Expected fragment sizes of the DNA bands from the F200Y, F167Y and E198A SNPs of *A. lumbricoides β-tubulin* isotype 1 gene after digestion with the appropriate restriction enzymes according to Zuccherato LW et al.,^22^ Furtado LFV et al^21^ and Rashwan N et al.^25^.

Codon	Restriction enzyme	Genotype	Fragment sizes (bp)
**F167Y** TTC, TTT/ Phenylalanine → TAC, TAT/Tyrosine	*RsaI*	Homozygous wild-type genotype (TTC/TTC**)**	543 bp and 65 bp
		Heterozygous variant genotype (TTC/TAC)	543 bp, 404 bp, 139 bp and 65 bp
		Homozygous variant genotype (TAC/TAC)	404 bp, 139 bp and 65 bp
**F200Y** TTC/Phenylalanine → TAC/Tyrosine	*HpyCH4III*	Homozygous wild-type genotype (TTC/TTC**)**	92 bp
		Heterozygous variant genotype (TTC/TAC)	92 bp and 181 bp
		Homozygous variant genotype (TAC/TAC)	181 bp
**E198A** GAG, GAA/Glutamic acid → GCG, GCA/Alanine	*BsmI*	homozygous wild-type genotype (GAA/GAA)	608 bp
		heterozygous variant genotype (GAA/GCA)	608 bp, 500 bp and 108 bp
		The homozygous variant genotype (GCA/GCA)	500 bp and 108 bp

### Statistical analysis

Data was analysed using STATA version 13 statistical software package (STATA Corp LLC, Lakeway Drive, TX, USA). A p-value < 0.05 was considered statistically significant.

## Results

### Participants demographics, deworming history and helminth status

The median age and BMI of the study population was 37.5 years and 24.5 kg/m^2^, respectively and there were an equal distribution of males and females. The Kato-Katz technique, which is the “gold standard” for the enumeration and detection of helminth eggs and worms from stool samples, proved to be the most sensitive method in detecting *A. lumbricoides* infections in all 20 stool samples whereas the Mini Parasep technique only detected infections in 13 of the 20 stool samples analysed (65 %). Regarding deworming, 3 of the 20 participants had previous exposure to deworming medication in the past 6-months, and 5 of the 20 participants did deworming every 6 to 12 months in their households (Table 3).

**Table 3:** Demographic characteristics and coproscopy results for all participants (*n* = 20).

Age (years)	Gender (M/F)	BMI (kg/m^2^)	Kato-Katz Results	Mini Parasep Results	Kato-Katz *A. lumbricodes* egg count (eggs per gram of stool)	Previous exposure to adeworming medication in the past 6-months.	How often is deworming done in household	Who gets deworming treatment in the household
30	F	36.9	*A. lumbricoides*	*A. lumbricoides*	169	No	Once a year	Children only
18	M	17.8	*A. lumbricoides*	*A. lumbricoides*	265	No	Never	None
46	M	23.7	*A. lumbricoides*	No parasite detected	409	Yes	Never	None
34	M	20.8	*A. lumbricoides* and *Taenia* spp.	*A. lumbricoides* and *Taenia* spp.	242	No	Never	Children only
42	F	23.8	*A. lumbricoides*	*A. lumbricoides*	745	Yes	Once in 6 months	Everyone
42	M	30.7	*A. lumbricoides* and *Trichuris Trichiura*	*A. lumbricoides* and *Trichuris Trichiura*	4	No	Never	None
47	M	31	*A. lumbricoides*	*A. lumbricoides*	25	No	Never	None
65	M	25.4	*A. lumbricoides*	*A. lumbricoides*	49	No	Never	None
51	F	49.8	*A. lumbricoides*	No parasite detected	24	Yes	Unsure	Children only
37	F	18.3	*A. lumbricoides* and *Hookworm* spp.	*A. lumbricoides* and *Hookworm* spp.	25	No	Never	None
58	F	38.2	*A. lumbricoides*	*A. lumbricoides*	146	No	Once in 6 months	Children only
35	F	25	*A. lumbricoides*	No parasite detected	24	No	Never	None
24	M	26	*A. lumbricoides*	No parasite detected	1	No	Unsure	None
35	M	21	*A. lumbricoides*	*A. lumbricoides*	121	No	Unsure	None
34	M	17	*A. lumbricoides*	*A. lumbricoides*	25	No	Never	None
36	F	24	*A. lumbricoides* and *Taenia* spp.	No parasite detected	4	No	Unsure	None
43	F	40	*A. lumbricoides*	No parasite detected	49	No	Once in 6 months	Children only
45	F	21	*A. lumbricoides*	No parasite detected	48	No	Never	None
36	M	25	*A. lumbricoides*	*A. lumbricoides*	20	No	Once in 6 months	None
38	M	20	*A. lumbricoides*	*A. lumbricoides*	194	No	Unsure	None

a Mebendazole and Albendazole are the two Benzimidazoles registered in South Africa for deworming.

### Use of benzimidazoles in south africa

Mebendazole is available over the counter as self-medication while Albendazole is a schedule 4 prescription medicine. Mebendazole is also used for the school-based mass deworming programme initiated in 2016 in South Africa^11^ The latter drug is also prescribed to adults presenting at primary health care clinics who are diagnosed with worm infections, albeit only a small proportion of adults are screened for worm infections in health facilities in South Africa, as this is not a routine practice. Only in cases where a clinician has a high index of suspicion for worm infection, then a request for a laboratory screening is requested. As such, the use of deworming medication among adults may be prohibited by cost of the drugs for over the counter self-medication.

### Verification and sequencing of the β-tubulin isotype 1 gene of A. lumbricoides positive worm extracts

**Fig. 1:**
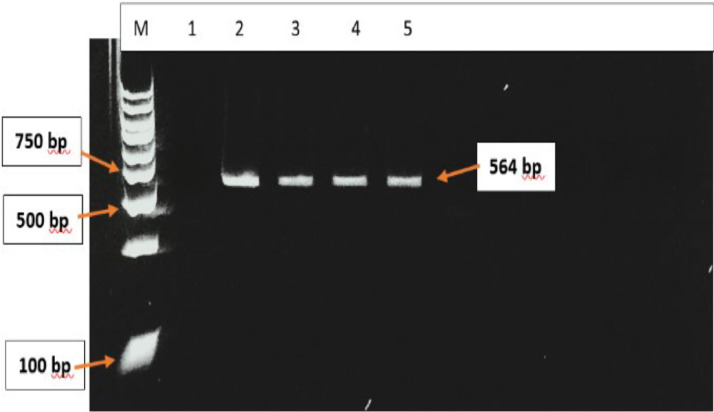
Agarose gel electrophoresis image of the *β-tubulin* isotype 1 gene of *A. lumbricoides* from the four positive control worm samples after RT-PCR. Lane M contains the 100 bp molecular weight marker, Lane 1 contains the negative control, lanes 2 – 5 contains the *β-tubulin* isotype 1 gene (564 bp) positive samples.

**Fig. 2:**
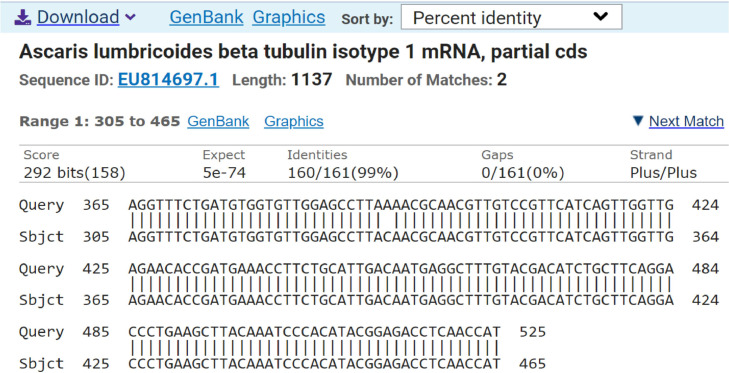
Sanger sequencing of the positive control PCR amplicons of the *β-tubulin* isotype 1 gene of *A. lumbricoides* worm samples showing a 99 % sequence match (accession number EU814697.1) and sequence alignment to the β-tubulin isotype-1 mRNA, partial CDS of *A. lumbricoides* on NCBI BLAST.

The *β-tubulin* isotype 1 gene of *A. lumbricoides* worm extracts were successfully amplified. The expected band size of 564 bp[21,22,25,26] was observed with agarose gel electrophoresis (Fig. 1). Sanger sequencing and sequence analyses performed on the PCR amplicons verified the *β-tubulin* isotype 1 gene and the absence of the F200Y, E198A and F167Y SNPs. The PCR positive control amplicons were identified with a 99 % sequence match (accession number EU814697.1) and sequence alignment to the *β-tubulin* isotype 1 mRNA (Fig. 2) using NCBI blast. The DNA isolated from stool eggs of 20 infected participants was also sent for sequencing however they did not detect the *β-tubulin* isotype 1 gene.

### Sequencing of the β-tubulin isotype 1 gene from A. lumbricoides egg positive stool samples

Sanger sequencing results for the *β-tubulin* isotype 1 gene from *A. lumbricoides* egg positive stool samples (*n* = 20) did not identify the *β-tubulin* isotype 1 gene. Thirteen of the sequenced samples did not match with any results on NCBI BLAST, and 7 of the sequenced samples partially matched with other bacterial and mammalian species (Supplementary Table S1).

### Detection of the β-tubulin isotype 1 gene SNPs (F200Y, E198A and F167Y) of A. lumbricoides using RFLP-PCR

**Fig. 3:**
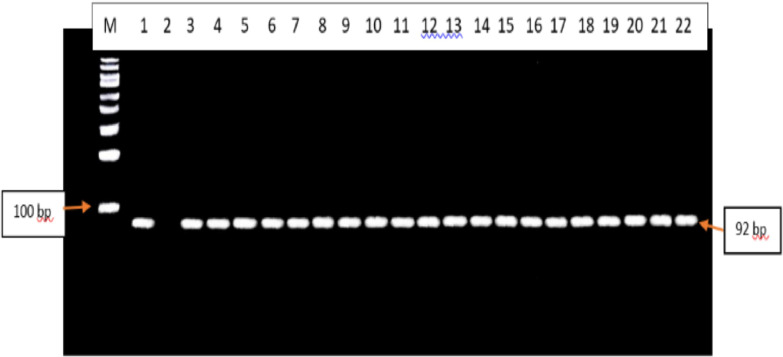
Agarose gel electrophoresis image of the F200Y codon in the *β-tubulin* isotype 1 gene of *A. lumbricoides* after RFLP-PCR. Lane M contains the 100 bp molecular weight marker, lane 1 contains the positive control sample, lane 2 contains the negative control, lanes 3 – 22 contains the 92 bp F200Y RFLP-PCR products that are observed to be the homozygous wild-type genotypes (T/T genotype).

**Fig. 4:**
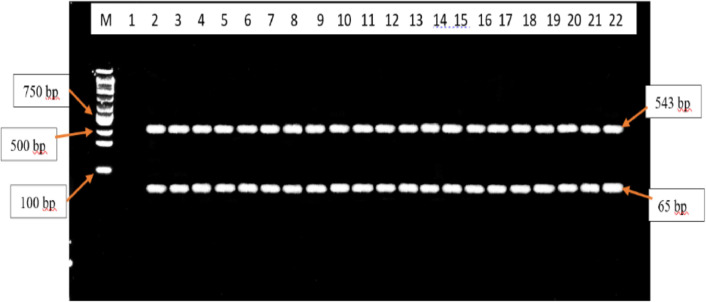
Agarose gel electrophoresis image of the F167Y codon in the *β-tubulin* isotype 1 gene of *A. lumbricoides* after RFLP-PCR. Lane M contains the 100 bp molecular weight marker, lane 1 contains the negative control sample, lane 2 contains the positive control sample, lanes 3 – 22 contains the 543 bp and 65 bp F167Y RFLP-PCR products that are observed to be the homozygous wild-type genotype (T/T genotype).

**Fig. 5:**
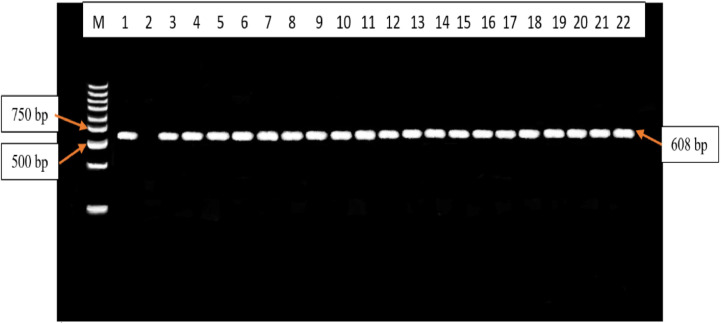
Agarose gel electrophoresis image of the E198A codon in the *β-tubulin* isotype 1 gene of *A. lumbricoides* after RFLP-PCR. Lane M contains the 100 bp molecular weight marker, lane 1 contains the positive control sample, lane 2 contains the negative control sample, lanes 3 – 22 contains the 608 bp E198A RFLP-PCR products that are observed to be the homozygous wild-type genotype (A/A genotype).

The RFLP-PCR products analysed did not show any evidence of SNPs at codons F200Y, E198A and F167. All the F200Y RFLP-PCR products produced a single band of 92 bp in size. Therefore, all samples were the homozygous wild-type genotype (T/T genotype)[25] (Fig. 3). All the F167Y RFLP-PCR products produced 2 bands of 543 bp and 65 bp in size. Therefore, all samples were the homozygous wild-type genotype (T/T genotype)[22] (Fig. 4). All the E198A RFLP-PCR products produced a single band of 608 bp in size. Therefore, all samples were the homozygous wild-type genotype (A/A genotype)[22] (Fig. 5).

## Discussion

STHs pose a significant public health concern, particularly among school-aged children where current benzimidazole Mass Drug Administration (MDA) programmes are ongoing, creating the need for surveillance systems to monitor drug resistance^28^ South Africa initiated the mass drug administration to school-going children in February 2016, using 500 mg Mebendazole^11^ This current study aimed to detect the prevalence of SNPs associated with potential benzimidazole resistance at codons F167Y,^20^ F200Y[21] and E198A[21,22] in the *β-tubulin* isotype 1 gene of *A. lumbricoides* from stool DNA extracts in a South African adult sample population. Using both Sanger sequencing and RFLP-PCR, the *β-tubulin* isotype 1 gene was detected in the positive worm extract. Although sequencing of the *β-tubulin* isotype 1 gene could not be confirmed from egg positive stool samples, the RFLP-PCR confirmed the presence of the homozygous wild-type genotypes for each SNP. In both the worm extract and egg positive stool samples, the F167, F200Y and E198A SNPs were not detected.

The inability of the Sanger sequencing method to detect the *β-tubulin* isotype 1 gene directly from egg positive stool samples may be attributable to inhibitory impurities from the stool. Sequencing the purified worm extract proved successful. This is in line with previously reported studies which initially purified the worms/eggs from stool before DNA extraction^12^^,^^21^^,^^22^^,^^25^^,^^26^^,^^29^, ^30^, ^31^ These results further confirm that sequencing requires high quality, pure nucleic acids. As such, the use of DNA extracted directly from stool samples to detect the SNPs is only feasible through RFLP-PCR as shown by the current study.

No benzimidazole resistant associated SNPs at codons F200Y, E198A and F167Y were observed in the present study. Studies reported on the absence of these SNPs in the *β-tubulin gene* family of *Ascaris lumbricoides* in Africa: Kenya,^29^ Zanzibar,^29^ Uganda,^29^ Tanzania,^12^ Ethiopia[12] and Rwanda;[30] the Americas: Panama,^29^ Brazil[22] and Honduras;[31] and Europe: Belgium^12^ However, most of the reported studies used school children. Our study used adults (18-years and above) due to South Africa being the last country to implement MDA programmes using Albendazole or Mebendazole to school aged children therefore, having comparatively less exposure to the drugs. Although no mutations were observed in this present study, and the studies mentioned above, this does not exclude the possibility of these mutations occurring in future or in other *A. lumbricoides* endemic countries.

The potential development of resistance to benzimidazole drugs in human is a serious threat to current MDA programs implemented globally^32^ Benzimidazole drugs act by disrupting microtubule formation which is essential for parasite motility and survival^33^ The *β-tubulin* protein is a core component of microtubules and is the primary target of benzimidazoles. Mutations in the *β-tubulin* gene can alter the binding affinity of benzimidazoles, leading to resistance^33^ Jones et al^13^ showed that in the *β-tubulin* gene family of the *Ascaris* genus, E198A is a crucial amino acid for benzimidazole binding of *β-tubulins* and the E198A and F200Y SNPs both confer resistance by disrupting this key anchor point however, they did not observe any effect of the F167Y mutation^13^ In veterinary nematodes benzimidazole resistance is caused by a SNP in the *β-tubulin* isotype 1 gene at codon positions F200Y, F167Y or E198A. These SNPs have already been observed in *Numenius americanus, T. trichiura* and *Haemonchus* contortus^25^^,^^29^^,^^34^ Additionally, in humans, correlation of these SNPs with poor response to benzimidazole treatment has been reported for the *T. trichiura* species*.* Rashwan and colleagues[25] found the frequency of the E198A and F167Y SNPs increased significantly in individuals who responded poorly to Albendazole^25^^,^^29^

The first signs of reduced susceptibility of *A. lumbricoides* to benzimidazole treatment was observed by Krücken et al^30^ in Rwandan school children, indicated by the calculated faecal egg count reductions being less than 95 % indicating a reduced efficacy to treatment however, they did not find any SNPs at codons F167Y, F200Y and E198A^30^ This correlates with the findings in this present study where all genotypes observed at codons E198A, F167Y and F200Y were 100 % homozygous wild-type genotypes. This data suggests that although mutations in codons F200Y, E198A and F167Y were not observed in the current study, development of anthelmintic resistance cannot be ruled out based on the small sample size and therefore requires further investigation to determine the factors that influence benzimidazole resistance in *A. lumbricoides* especially in highly endemic areas where MDAs are ongoing^30^

Matamoros et al^31^ investigated the prevalence of the F167Y, E198A and F200Y SNPs in the *β-tubulin gene* of 40 genomic sequences from *A. lumbricoides* samples obtained from Honduran children 0.6–13 years of age, that had received prior treatment with Albendazole. It was noted that no mutations at codons F200Y, F167Y and E198A were detected^31^ These results also agree with the findings in this present study.

Similar to the results observed in this present study, Zuccherato et al^22^ were also unsuccessful in detecting the presence of any mutations at codons E198A and F167Y in the *β-tubulin gene* family of *A. lumbricoides* samples from six Brazilian states^22^ Diwara et al^29^ developed a pyrosequencing assay to detect for a SNP at codon F200Y in the *β-tubulin gene* family of *A. lumbricoides* samples from Zanzibar, Uganda and Panama. No SNP was detected^29^

Roose et al^12^ observed at least seven different *β-tubulin* genes in *A. lumbricoides* samples from Ethiopia, Tanzania and Belgium, as well as an eighth putative *β-tubulin* encoding gene. The *β-tubulin* isotype 1 gene and isotype 2 gene were shown to be highly expressed during the entire parasite life cycle and, therefore, are more likely to play an important part in benzimidazole resistance^12^ However, they were unable to detect any SNPs at codons F167Y and E198A in the *β-tubulin gene* family of *A. lumbricoides* from their sample population^12^ Much like the results observed in our present study, these studies were unable to detect SNPs at codons F200Y, E198A and F167Y in the *A. lumbricoides β-tubulin gene*[12,22,29,30,31] however, the molecular methods modified for SNP detection in these studies will prove to be valuable tools for future research^12^^,^^21^^,^^22^^,^^29^, ^30^, ^31^

By contrast, a study conducted by Diwara et al.,^26^ detected high frequencies of the F167Y SNP homozygous variant genotype (TAC/TAC) in the *β-tubulin gene* of *A. lumbricoides* prior to benzimidazole treatment in Haiti, Kenya and Panama^26^ Post-treatment samples from Panama and Kenya showed no significant reduction in the homozygous variant genotype (TAC/TAC) frequency^26^ However, in Haiti, post-treatment samples showed an increase in the homozygous variant genotype (TAC/TAC) frequency although the infections had cleared^26^ These observations suggest that the SNP at codon F167Y may not impact the efficacy of the drug, although more testing needs to be done to confirm this. Similarly, to this present study, all the genotypes observed by Diwara et al^26^ were 100 % homozygous wild-type genotype at codons F200Y (TTC/TTC**)** and E198A (GAA/GAA).

Additionally, Rashwan and colleagues[25] developed a SmartAmp2 method that targeted polymorphisms in the *β-tubulin* isotype 1 gene of *A. lumbricoides.* They detected the SNP TTC>TAC in codon F167Y in Haiti. Consequently, for *A. lumbricoides*, the existing data is not yet well-defined for the use of these SNPs as a marker for benzimidazole resistance^25^ The first report of the F200Y SNP in the *β-tubulin* isotype 1 gene of *A. lumbricoides* was made by Furtado et al^21^ They observed a mutation at codon F200Y at 0.5 % frequency (*n* = 4/854)^21^ Despite the low frequency observed, the presence of this SNP is indicative of the potential of these parasite populations to develop resistance to current treatments and possibly at higher levels in the near future^21^

### Limitations

Samples in this study were purposively selected on the basis of stool egg positivity thus introducing selection bias. By default, a small proportion of the total study population tested positive for *Ascaris lumbricoides* eggs in stool. Only twenty of the Ascaris egg positive samples were purposively selected due to budgetary constraints of laboratory testing, for the costly multiple molecular analyses. This small sample size therefore affects the representativeness of the results. As observed by Furtado et al.,^21^ only 4 out of 850 samples had a mutation at codon F200Y at 0.5 % frequency (*n* = 4/854)^21^ Therefore, screening for SNPs in a larger population sample size will provide a better representativeness of SNPs in our South African population. Furthermore, there is no reported studies in a South African population investigating the prevalence of the F200Y, E198A and F167Y SNPs of the β-tubulin isotype 1 gene in Ascaris lumbricoides. Hence, this pilot study and neglected field of research lays the groundwork for larger-scale surveillance studies.

We acknowledge the significance of genetic diversity/population structure analysis for monitoring regional drug resistance and gaining insight into the local adaptations and monitoring transmission dynamics in order to improve the control and eradication programmes. Due to the fact that: (i) The β-tubulin gene SNPs (F200Y, E198A and F167Y) that are commonly associated with resistance to Benzimidazole drugs were not detected in this study, and (ii) The small non-representative sample size collected in the focal area (South of Durban) would not reflect a diverse representative regional sample in this pilot study, we could not assess the overall genetic diversity or population structure of *A. lumbricoides*. However, we acknowledge the need for geographically diverse sampling in a wider area of the country in future studies.

The use of adults as participants in this study had its drawbacks considering only 15 % of the study population reported prior exposure to deworming treatment as opposed to children that have received multiple exposures to Benzimidazole treatments due to ongoing deworming MDA programmes. Thus, the selection pressure that is introduced by multiple exposures to Benzimidazole treatments for the development of resistance is unknown in our adult sample population compared to the school children who have received multiple doses of the drug over the years. It is also noted that the history of deworming in this study population may also suffer recall bias from the self-reported approach for collecting this data using structured questionnaires.

Lastly, the present study did not generate any phenotypic resistance data since the aim was solely to investigate the prevalence of the F200Y, E198A and F167Y SNPs of the *β-tubulin* isotype 1 gene in *Ascaris lumbricoides* isolated from a South African population. However, the authors do acknowledge that phenotypic resistance in *Ascaris lumbricoides*, particularly to Benzimidazole drugs like albendazole and mebendazole, is a complex issue with multiple potential contributing factors. While some studies have focused on mutations in the *β-tubulin* isotype 1 gene (F200Y, E198A and F167Y), which are known to cause resistance in other nematodes,^14^, ^15^, ^16^^,^^19^^,^^20^ these mutations have not been consistently found in *Ascaris lumbricoides*^21^^,^^22^^,^^24^, ^25^, ^26^ This suggests that resistance mechanisms in *Ascaris lumbricoides* may involve other factors, such as: (i) Changes in drug metabolism (increased activity of cytochrome P450s or Glutathione S-Transferases [GSTs] which could degrade benzimidazoles),^35^ (ii) Altered drug uptake or efflux (overexpression of P-glycoproteins [P-gp] and other multidrug ATP-Binding Cassette [ABC] transporters may expel benzimidazole before the latter can act),^36^ (iii) Target Site Copy Number Variation (gene duplication of *β-tubulin* may dilute drug effects, as observed in some nematodes),^37^ and (iv) Increased stress responses (enhanced Heat Shock Proteins [HSP] activities; HSP70 and HSP90 require ATP to function and are upregulated during Benzimidazole drug exposure and thermal stress, while small HSPs [sHSPs] do not require ATP to function and protect against drug-induced oxidative damage)^38^

To assess for phenotypic resistance, it is important to isolate and grow *Ascaris lumbricoides* eggs, larvae and worms from infected individuals, and thereafter conduct *in vitro* studies. *In vitro* methods used to detect phenotypic resistance include: (i) Larval Development Assays (eggs or larvae are exposed to increasing Benzimidazole drug concentrations, and development inhibition is measured). Microfluidic assays can track real-time larval motility under Benzimidazole drug pressure, (ii) Adult worm motility assays (worms are exposed to benzimidazole drugs, and paralysis/death is quantified using the Worminator system for movement analysis), (iii) Dye-based viability assays (MTT assay used to measure metabolic activity, whereby resistant worms retain dye reduction capacity), and (iv) Egg Hatch Assay (EHA) (eggs are exposed to drug; resistant eggs hatch despite treatment). Human studies can also be utilized to detect phenotypic resistance. The Fecal Egg Count Reduction Test (FECRT) can be used to conduct egg counts in individuals pre-treated and post-treated with benzimidazole drugs.

## Conclusion

In this present study, all genotypes observed at codons F200Y, E198A and F167Y were the homozygous wild-type genotype (100 % frequency). The RFLP-PCR method used for the analysis and identification of genotypes at codons where possible SNPs that are associated with potential benzimidazole resistance in the *β-tubulin* isotype 1 gene of *A. lumbricoides* proved to be a successful alternative compared to conventional Sanger sequencing. Although there were no SNPs observed at codons F200Y, E198A and F167Y in the *β-tubulin* isotype 1 gene of *A. lumbricoides,* these findings in this present study agree with previous studies published in Africa, the Americas and Europe^12^^,^^22^^,^^29^, ^30^, ^31^ The observations made in the small target population of this present study does not exclude the possibility of the occurrence of SNPs at codons F167Y, E198A and F200Y associated with potential benzimidazole resistance in the *β-tubulin gene* family of *A. lumbricoides* as seen in other studies[21,25,26] and in other veterinary nematodes^25^^,^^29^^,^^33^ In addition to this, a reduced susceptibility of *A. lumbricoides* to benzimidazole has been observed by Krücken et al^30^ therefore, it is necessary to monitor current MDA programmes of benzimidazole for *A. lumbricoides* especially in highly endemic countries and screen for SNPs associated with potential benzimidazole resistance in the *β-tubulin gene* family of *A. lumbricoides.* The characterization and detection of SNPs associated with potential benzimidazole resistance in the *β-tubulin* isotype 1 gene of *A. lumbricoides* will allow deeper insight into the susceptibility of *A. lumbricoides* to current treatment regimens using Albendazole and Mebendazole. This could propagate further studies on alternative methods of treatment for *A. lumbricoides.* Currently, there is a lack of data on the circulating genotypes of *A. lumbricoides* SNPs in the *β-tubulin* isotype 1 gene of *A. lumbricoides* in South African populations that are susceptible to benzimidazole resistance, particularly in the under-developed areas South of Durban, KwaZulu-Natal, South Africa. Identification of the SNPs that are associated with potential benzimidazole resistance will provide insight into the molecular epidemiology of *A. lumbricoides* in this region and its susceptibility to benzimidazole drugs which can be used as a foundation for alternate treatment methods for *A. lumbricoides*.

## Data availability

Data from this study is available from the corresponding author upon reasonable request.

## Ethical clearance

All ethical standards for research were followed in this article without direct contact with animal or human subjects. Ethical clearance for this study was given by the Biomedical Research Ethics Committee (BREC) at the University of KwaZulu-Natal (BREC Ref n° BE351/19 and BREC/00,004,834/2022).

## Funding

Research reported in this publication was supported by the South African Medical Research Council (SAMRC) (ZLMK MSC grant number: HDID5149/KR/202) through its Division of Research Capacity Development under the Research Capacity Development Initiative from funding received from the South African National Treasury. The content and findings reported/illustrated are the sole deduction, view, and responsibility of the researchers and do not reflect the official position and sentiments of the SAMRC.

## Conflicts of interest

The authors declare no conflicts of interest.

## Appendix Supplementary materials

Image, application 1
