# Supplementary material for: Single nucleotide polymorphisms associated with benzimidazole resistance of the β-tubulin isotype 1 gene in Ascaris lumbricoides isolated in South Africa
Source: Braz J Infect Dis. 2025 Jun 28;29(5):104556. doi: 10.1016/j.bjid.2025.104556 (PMC12266506; doi:10.1016/j.bjid.2025.104556)
Supplement: Supplementary file 1 [file mmc1.docx]

**BJID-D-24-00383_Supplementary Material**

**Supplementary Table S1** Sanger sequencing of the seven *A. lumbricoides* positive stool sample PCR amplicons showing partial sequence matches to other bacterial and mammalian species on NCBI BLAST.

| ***A. lumbricoides* positive stool sample PCR amplicon** | **Sequence match (%)** | **Accession number** | **Bacterial Species** |
| --- | --- | --- | --- |
| 1 | 71.03 | WP330618835.1 | *Mogibacterium* *sp*. |
| 2 | 88.66 | CP048433.1 | *Collinsella aerofaciens* |
| 3 | 87.69 | AP022868.1 | *Bifidobacterium longum* |
| 4 | 89.63 | AP022868.1 | *Bifidobacterium longum* |
| 5 | 92.63 | AP022868.1 | *Bifidobacterium longum* |
| 6 | 88.89 | XM003272206.3 | *Nomascus leucogenys* |
| 7 | 98.18 | BK039054.1 | *Bacteriophage sp.* |
